# Editorial: Contemporary challenges in immunologic testing in clinical and research laboratories

**DOI:** 10.3389/fimmu.2023.1259823

**Published:** 2023-07-27

**Authors:** Luis Eduardo C. Andrade, Edward K. L. Chan, Stefan Vieths, Pablo Engel, Michael Kirschfink

**Affiliations:** ^1^ Rheumatology Division, Escola Paulista de Medicina, Universidade Federal de São Paulo, São Paulo, Brazil; ^2^ Immunology Division, Fleury Group Medicine and Health Laboratories, São Paulo, Brazil; ^3^ Department of Oral Biology, University of Florida, Gainesville, FL, United States; ^4^ Division of Allergology, Paul-Ehrlich-Institut, Langen, Germany; ^5^ Department of Biomedical Sciences, University of Barcelona, Barcelona, Spain; ^6^ Institute of Immunology, University of Heidelberg, Heidelberg, Germany

**Keywords:** immunology testing, autoimmune diseases, allergy testing, complement system, cluster of differentiation (CD), immunoassays, quality assessment and standardization

Immunologic testing is an integral part of several areas related to immunology, embracing basic and applied research, clinical laboratory routine, epidemiological survey, blood bank control, and *in vitro* diagnostic industry (IVD) research, development, and production, just to mention a few. The complex network of the immune system, modeled by myriad soluble and surface molecules and multiple circulating and resident cells, reflects the great variety of “immunologic analytes” to be determined in the various immunologic tests addressing the diverse areas in which immunology plays a relevant role. These encompass a broad spectrum spanning several medical specialties, including allergic and autoimmune diseases, primary and secondary immunodeficiencies, infectious diseases, cancer, vaccination, and epidemiology. Aside from immune-related diseases, immunoassays are also crucial tools in most areas of medicine, from endocrinology to toxicology, as exemplified by immunoassays for the determination of hormones, therapeutic drugs, serum proteins, vitamins, and tumor biomarkers, among others.

Standardization and quality assessment are crucial for any laboratory analysis so that results obtained in different laboratories and different parts of the world share a minimum degree of coherence. Each analyte to be determined has peculiar characteristics that affect the respective laboratory assay and, consequently, affect the way these assays need to be standardized and controlled. The myriad analytes addressed in immunologic testing display multiple peculiarities, rendering standardization and quality assessment in immunology a complex and multifaceted field. Some molecules do not show relevant polymorphism, such as C-reactive protein, soluble IL-2 receptor, and complement factor C1q. In contrast, some other targets of immunology testing represent the most polymorphic elements in biology, such as the major histocompatibility complex genes and ensuing proteins. Cytokines and several complement components are extremely labile, requiring specific pre-analytical handling, whereas immunoglobulins are rather stable at room temperature for several hours. Samples for cryoglobulin determination must be handled at 37°C during the entire pre-analytical stage because these peculiar immunoglobulins may precipitate, becoming trapped in the blot clot, which would yield false negative results. These are just a few examples of the particularities of immunologic analytes that influence the standardization of immunologic assays.

A substantial branch of immunology testing refers to the determination of antibodies specific to a certain target, be it a microorganism, an autoantigen, an allergen, an alloantigen, or a toxin. In fact, these assays are set to determine the humoral immune response to a given antigen and this is not represented by a monoclonal antibody, but rather by a polyclonal collection of antibodies that share that antigen as their target. Considering the polymorphism of the immunoglobulin genes and the random dynamics of the development of the antibody response, it is obvious that each individual forms a distinctive collection of antibodies against each antigen. The mosaic of antibodies in each individual is analogous to a “fingerprint” characterized by different proportions of antibodies with different isotypes, targeted epitopes, avidities, and Fc post-translational modifications (glycosylation, acetylation, etc.), all these being balanced at different serum concentrations. In a sense, the panel of anti-X antibodies in individual A will be necessarily different from the panel of anti-X antibodies in individual B. Under this perspective, it is easy to realize that any given immunoassay to determine anti-X antibodies will perform differently for different individuals, and different immunoassays for anti-X antibodies can yield different results in the same sample. In fact, in contrast to simple analytes (all molecules are the same across individuals) such as glucose and C-reactive protein, antibodies are complex analytes (each individual has its own array of molecules) that represent the functional response of the humoral immune system against a given antigen. This scenario brings a considerable challenge for the IVD industry in developing products that perform appropriately for a relevant part of the population of interest. However, the biggest challenge is the standardization and harmonization of proprietary immunoassays of dozens of IVD industries originated in different parts of the world, calibrated, and validated using samples from patients from diverse ethnic and environmental backgrounds.

In order to handle the challenge of standardization in immunology testing, the International Union of Immunology Societies (IUIS) has established a committee dedicated to Quality Assessment and Standardization (QAS) in Immunology. The QAS Committee operates for over four decades by means of specific subcommittees, namely, the Allergen Standardization Subcommittee (1), the Autoantibodies in Rheumatic and Related Diseases Subcommittee (2), the Complement Subcommittee (3), the Leukocytes Subcommittee (4, 5, www.hcdm.org), and the Big Data in Immunology subcommittee (https://iuis.org/committees/qas/big-data-for-immunology-sub-committee/). Each of these subcommittees coordinates various actions aiming to promote quality assessment and standardization in their respective field. These actions include the preparation and distribution of reference materials (standards), the establishment of guidelines and policies, and educational activities. The Research Topic *Contemporary challenges in immunologic testing in clinical and research laboratories* is a recent initiative from the QAS Committee and addresses several aspects of interest in the area.

Serological immunoassays for the diagnosis of infectious diseases have been a major priority in research, IVD industry, and clinical laboratories. Although this activity has been flourishing for decades, the recent Severe Acute Respiratory Disease Coronavirus 2 (SARS-CoV-2) pandemic has brought to spotlight the crucial role of serologic immunoassays in the management of infectious diseases. In the early days of the pandemic, robust and reliable serological immunoassays should be promptly developed to characterize the abundance, neutralization efficiency, and duration of antibodies associated with the humoral immune responses to SARS-CoV-2. In addition to the use of these tests for the management of individual patients, the accurate detection, measurement, and characterization of the anti-SARS-CoV-2 humoral response (i.e., temporal dynamics, isotype distribution, neutralization capacity) has been critical for vaccine development, establishment of guidelines for healthcare and at-risk workers, and monitoring reinfections with genetic variants of the virus. All these aspects were brilliantly covered in this Research Topic by Galipeau et al. who also address the benefits and limitations of the currently available commercial and laboratory-based serological assays, in addition to the potential of cross-reactivity and possible immunological back boosting by seasonal coronaviruses.

The urgent need for a low-cost assay to diagnose dengue efficiently is addressed in the manuscript by Lai et al. This is especially relevant since no commercial dengue antigen tests able to differentiate viral serotypes are available. The authors have developed a multiplex lateral flow immunoassay (LFIA) that can identify mono- and co-infection of different serotypes of dengue viruses in mosquitoes. This new assay provides a simple tool for the rapid detection of dengue and is efficient for the differential diagnosis of fever patients in regions where medical resources are limited.

Another area of great contemporary interest is the field of immunobiological drugs embracing monoclonal antibodies and fusion proteins targeting key elements of the immune system with the aim of modulating and controlling inflammatory and autoimmune disorders. Initiating in the mid-1990s, this therapy modality has proven to be able to change the natural history of a host of chronic and disabling diseases such as rheumatoid arthritis, ankylosing spondylitis, Crohn’s disease, neuromyelitis optica, just to cite a few (6). A plethora of monoclonal antibodies and their respective molecular targets is currently part of the routine jargon of physicians and patients and the area is in frank expansion. Lately, several of the original monoclonal antibodies have been licensed to be produced as biosimilar drugs. In parallel, the concept of therapeutic drug monitoring has been established with the aim of achieving the most appropriate drug serum levels and optimizing the therapeutic results. This scenario clearly indicates an urgent need for harmonization and standardization of the original immunobiological drugs and their biosimilar correlates with respect to pharmacokinetics and bioactivity. One key element for standardization in the field is the establishment of International Standards (IS) for each monoclonal antibody. In this Research Topic, Wadhwa et al. originally present the first World Health Organization IS for adalimumab, a leading anti-TNF-α monoclonal antibody. This IS will have great utility in a wide range of applications, including the validation, calibration, and standardization of bioassays for measuring adalimumab and biosimilar effectivity, as well as immunoassays to determine adalimumab/biosimilar serum levels in therapeutic drug monitoring.

The screening for autoantibodies using the indirect immunofluorescence assay on HEp-2 cells (HEp-2 IFA) is widely used in the diagnostic investigation of patients suspected of systemic autoimmune diseases. The immunofluorescence pattern elicited by reactive samples is very useful because it provides indirect information on the probable antigenic targets of the autoantibodies in the sample. This topic has been largely developed by the International Consensus on ANA Patterns (ICAP) initiative (7, 8, www.anapatterns.org). In this Research Topic, Röber et al. present an international multicenter study establishing a novel HEp–2 IFA pattern strongly associated with autoantibodies to SS–A/Ro 60kDa, an autoantibody observed in patients with systemic lupus erythematosus and Sjögren’s syndrome.

Dozens of competent IVD industries offer convenient kits with slides containing fixed HEp–2 cells and all the reagents necessary for the HEp–2 IFA procedure. It has been demonstrated that the HEp–2 IFA pattern produced by a given sample may vary according to the conditions used to cultivate and fix the cells (9). In this Research Topic, Silva et al. provide an extensive analysis of the HEp–2 IFA pattern observed in four high–ranked HEp–2 IFA kits using 900 samples from individuals with an array of clinical conditions. They found that non–reproducibility of the HEp–2 IFA pattern is rather prevalent and occurs more frequently in samples with weaker reactivity (lower titer) as well as in some specific patterns (e.g., nucleolar patterns). In addition, HEp–2 IFA–reactive samples from healthy individuals tended to present non–reproducibility of results among HEp–2 IFA kits more often than samples from patients with systemic autoimmune diseases (Silva et al.). The non–reproducibility phenomenon demonstrated by Silva et al. should have an important impact on the clinical use of the HEp–2 IFA test and, therefore, international initiatives are needed to promote the harmonization of the properties and performance of HEp–2 IFA commercial kits.

Recent developments in modern complement analysis have been addressed by Frazer–Abel et al. Dysregulation and over–activation of the complement system are major causes of a variety of inflammatory and autoimmune diseases ranging from nephropathies, age–related macular degeneration (AMD), and systemic lupus erythematosus (SLE) to graft rejection, sepsis, and multi–organ failure. The clinical relevance of the complement system to immunologic diseases is reflected by the recent development of multiple drugs targeting complement with a broad spectrum of indications. The recognition of the role of complement in diverse diseases and the advent of complement therapeutics has increased the number of laboratories and suppliers entering the field. This has highlighted the need for reliable complement testing. The relatively rapid expansion in complement testing has presented challenges for a previously niche field. This is exemplified by the issue of cross–reactivity of complement–directed antibodies and by the challenges of the poor stability of many of the complement analytes, esp. of complement activation products. The complex nature of complement testing and increasing clinical demand has been met in the last decade by efforts to improve standardization among laboratories. Initiated by the *IUIS/ICS (International Complement Society) Committee for the Standardization and Quality Assessment in Complement Measurements*, 14 rounds of external quality assessment since 2010 resulted in improvements in the consistency of testing across participating institutions while extending the global reach of the efforts to meanwhile more than 300 laboratories in 30 countries. Worldwide trends of assay availability, usage, and analytical performance are summarized based on the experience from recent years. Progress in complement analysis has been facilitated by the quality assessment and standardization efforts that now allow complement testing to provide a comprehensive insight into deficiencies and the activation state of the system. This in turn enables clinicians to better define disease severity, evolution, and response to therapy.

Dysregulation of the complement system also contributes to the pathogenesis of preeclampsia, which is mainly characterized by gestational hypertension, proteinuria, systemic endothelial cell activation, and inflammatory overreaction. In search for appropriate biomarkers, Liu et al. investigated the levels of adipsin, C3a, C5a, and soluble endoglin (sENG) before delivery to assess their role in preeclampsia. Then, a follow–up analysis was conducted to determine whether complement levels and sENG fluctuate with gestational age and whether plasma adipsin and related important circulating complement molecules can be used as an early–pregnancy predictor and potential diagnostic biomarkers of preeclampsia (Liu et al.). They found that adipsin is likely a novel plasma biomarker to monitor the increased risk of preeclampsia in early pregnancy. Moreover, the increased plasma levels of adipsin, C5a, and sENG before delivery may be associated with preeclampsia.

Recurrent angioedema without urticaria (AE) in its hereditary (HAE) or acquired (AAE) form is commonly misdiagnosed due to restricted access and availability of appropriate laboratory tests. HAE with C1 inhibitor defect (HAE–C 1–INH) is associated with quantitative and/or functional deficiency of this multifunctional regulator. Although this bradykinin–mediated disease results mainly from a disturbance in the kallikrein–kinin system, traditionally complement evaluation has been used for diagnosis. Diagnosis is established by nephelometry, turbidimetry, or radial immunodiffusion for quantitative measurement of C1 inhibitor, and chromogenic assay or ELISA has been used for functional C1–INH analysis. However, as reviewed by Grumach et al. in this Research Topic, a large group of patients present with similar clinical manifestations to HAE but without C1–INH defect and normal C4 (HAE–nlC1–INH). Although a causative mutation cannot be found in a considerable number of patients with HAE–nlC1–INH, new variants in several genes have been associated recently with this form of the disease, such as angiopoietin 1 gene, plasminogen, kininogen, myoferlin, and heparan sulfate 3–O–sulfotransferase 6 genes. These new mutations not only imply novel mechanisms and systems involved in the pathogenesis of HAE but also open the possibility for new biomarkers and treatment targets.

The interesting paper by Kužílková et al. deals with the problem of a lack of reproducible identification of leukocyte subsets. The authors describe the development of a flow cytometric procedure for quantitative expression profiling of surface antigens on blood leukocyte subsets, which is standardized across multiple research laboratories. This workflow, bioinformatics pipeline, and optimized flow panels enable the mapping of the expression patterns of Human Leukocyte Differentiation Antigen (HLDA)–approved mAb clones to cluster of differentiation (CD) markers, benchmarking new antibody clones to established CD markers, and defining new CDs in future HLDA workshops.

The Opinion article by Di Rosa et al. discussed advances in the field of T cell proliferation analysis. It challenges the well–established idea that Ki–67 per se is an ideal marker of T cell proliferation. They propose the use of a new Ki–67/DNA dual staining, or TDS assay, which represents a more reliable approach by which human peripheral blood can be used to reflect the dynamics of human lymphocytes, rather than providing mere steady–state phenotypic snapshots.

The broad range of immunologic tests performed in clinical and research laboratories is in frank expansion and affects most areas of medicine. Quality assessment and standardization in immunology testing is a fundamental aspect that meets several challenges elicited by the peculiar characteristics of several of the immunologic analytes to be determined. International organizations dedicated to promoting standardization and quality assessment in different areas of immunology testing contribute substantially to the progress in the area. The IVD industry provides a variety of commercial kits, contributing to the widespread availability of immunology testing in clinical and research laboratories in most parts of the world. However, the plethora of commercial kits available adds an exceptional challenge to the standardization of the tests. Although these commercial products are licensed by official regulatory agencies, there is no formal collaboration between these official agencies and the international quality assessment and standardization initiatives formed by specialists in each area. A tripartite collaboration involving the IVD industry, international specialists, and official regulatory agencies has the genuine potential to improve significantly the standardization and harmonization of immunology testing worldwide.

## Author contributions

All authors listed have made a substantial, direct, and intellectual contribution to the work and approved it for publication.
